# A correlational study of Greek therapists’ responses to sexual and erotic feelings during psychotherapy

**DOI:** 10.1192/j.eurpsy.2025.1506

**Published:** 2025-08-26

**Authors:** M. Lazaridou, I. Sofroni

**Affiliations:** 1 ICPS, Athens, Greece

## Abstract

**Introduction:**

Erotic and sexual feelings within therapy are complex phenomena which provide important information on aspects of both the client’s and the therapist’s relational self and hence bear great significance for the outcome of the therapy. The present correlational study examined Greek therapists’ responses to sexual and erotic feelings during psychotherapy.

**Objectives:**

The following hypotheses arising from the relevant literature were formulated so as to explore the above aims of the study empirically.

Hypothesis 1: Women therapists experience more negative feelings than males.

Hypothesis 2: There will be a statistically significant difference between males and females in experiencing gratification feelings.

Hypothesis 3: Therapists with psychodynamic training will experience lower levels of dysphoric feelings compared to therapists of other orientations.

Hypothesis 4: The proportion of psychodynamic therapists that disclose sexual and erotic feelings towards a client in supervision will be significantly higher than that of therapists of other orientations.

Since the present is the first conducted in the cultural environment of Greece and the first one correlating quantitative data from a psychometric test with handling of erotic and sexual feelings questions, the study will investigate in addition further questions correlating years of experience, sexual orientation, therapist age, with measures of emotional reactions.

**Methods:**

Over two months, 139 adult psychotherapists completed an anonymous online survey. The demographics and attitudes of psychotherapy participants toward sexual and erotic feelings were collected. The Therapists’ Attitude toward Sexual and Erotic Feelings Scale measured emotions.

**Results:**

Results showed that male therapists felt more enjoyment than females, who were more afraid of the erotic or sexual. When controlling for therapist age, gendered difference in terror disappears. CBT and integrative therapists, who scored high on Aversion, also experienced higher threat levels. The present investigation showed no correlation between psychotherapeutic orientation and therapist erotic or sexual disclosure to supervised clients. Additional secondary analysis was done.

**Conclusions:**

The findings of the study imply that therapists’ emotional reactions to sexual and erotic sensations in psychotherapy are multifaceted and relate to their professional and personal identities. Psychotherapeutic training, licensure, supervision, and therapist implications are examined.

**Disclosure of Interest:**

None Declared

